# On-going transmission of human onchocerciasis in the Massangam health district in the West Region of Cameroon: Better understanding transmission dynamics to inform changes in programmatic interventions

**DOI:** 10.1371/journal.pntd.0006904

**Published:** 2018-11-14

**Authors:** Didier Bakajika, Laura Senyonjo, Peter Enyong, Joseph Oye, Benjamin Biholong, Elizabeth Elhassan, Daniel Boakye, Ruth Dixon, Elena Schmidt

**Affiliations:** 1 Sightsavers, Accra, Ghana; 2 Sightsavers, Haywards Heath, United Kingdom; 3 Consultant Senior Entomologist, Kumba, Cameroon; 4 Sightsavers, Yaoundé, Cameroon; 5 Ministry of Public Health, Yaoundé, Cameroon; 6 Noguchi Memorial Institute for Medical Research, Accra, Ghana; RTI International, UNITED REPUBLIC OF TANZANIA

## Abstract

**Background:**

Massangam health district (HD), in the West Region of Cameroon, has received ivermectin mass drug administration (MDA) for 20 years, however there is evidence of continued high transmission of *Onchocerca volvulus*. In order to better understand the transmission dynamics in the HD and inform intervention strategies there is a need to delineate the boundaries of the suspected area of high transmission within the wider transmission zone.

**Methodology/Principal findings:**

Parasitological and entomological surveys were conducted to map out the breeding sites of *Simulium damnosum* and evaluate the prevalence of onchocerciasis in neighbouring communities, including Makouopsap sentinel community. Potential rapids were prospected for identification of *S*. *damnosum* larvae and black flies collected to determine infectivity rates. Adults were assessed for the presence of *O*. *volvulus* microfilariae through a skin snip biopsy and examined for the presence of nodules. Anti Ov-16 antibodies were tested for in children. Four perennial breeding sites were identified on the Rivers Mbam and Nja. Large number of flies were collected along the River Mbam, especially in the rainy season, with up to 955 flies per day, suggesting this river is a perennial source of black flies. A total of 0.8% of parous flies were infective across the study area. Parasitological studies provided evidence of high rates of infection in the sentinel community and three neighbouring communities, with 37.1% of adults microfilariae positive in Makouopsap. High Ov-16 seropositivity in children also provided evidence of recent on-going transmission. In comparison, communities sampled further away from the sentinel community and neighbouring breeding sites were much closer to reaching onchocerciasis elimination targets.

**Conclusions/Significance:**

This study provides evidence of a particular geographic area of high transmission in an approximate 12 km range around the sentinel community of Makouopsap and the neighbouring breeding sites on the River Nja. To eliminate onchocerciasis by 2025, there is a need to explore alternative intervention strategies in this area of high transmission.

## Author summary

The global goal is to eliminate onchocerciasis by 2025. However, not all areas are on track to do so, as is the case for Massangam health district (HD) in the West Region of Cameroon. A new strategy is required in order to accelerate efforts to reach elimination targets. This study aimed to better understand the transmission dynamics of *Onchocerca volvulus* in Massangam HD through the identification of black fly (*Simulium damnosum)* breeding sites, parasitological and entomological studies. The study was able to define an approximate 12 km area of high transmission encompassing four communities with very high levels of infection and evidence of on-going transmission in humans. This area is facilitated by perennial productive black fly breeding sites in the neighbouring rivers of the Nja and Mbam. Multi-faceted interventions are required to target the reservoir of infection facilitating transmission. We suggest the need for a new test and treat strategy in the area of high transmission (with a drug that kills the adult *O*. *volvulus* worm living in humans) and larviciding of the breeding sites of the black fly. In areas surrounding the area, increase distribution of ivermectin from annual to twice yearly distribution, whilst also using innovative strategies to improve compliance.

## Introduction

Onchocerciasis also known as “river blindness” is a tropical disease caused by the filarial nematode *Onchocerca volvulus*. It is transmitted to humans through the bite of an infected black fly of the genus *Simulium*, which mainly breed in fast flowing rivers and streams. The female worms produce microfilariae (mf) that migrate out of the nodules and circulate in the skin [1] causing clinical manifestations, including forms of dermatitis. The mfs can also enter the eyes, leading to inflammatory lesions (keratitis, chorio-retinitis), optic nerve atrophy and blindness [2].

Ivermectin is a safe and potent microfilaricide that has been used widely in onchocerciasis endemic areas [3]. Evidence shows that annual or semi-annual distribution of ivermectin to affected communities can eliminate transmission of *O*. *volvulus* in Africa, especially in meso and lower hyper-endemic areas [4–7]. As a result the World Health Organisation (WHO) has declared onchocerciasis as a target for elimination by 2025 [8].

Rapid Epidemiological Mapping of Onchocerciasis (REMO) surveys conducted in Cameroon in the early 1990s showed that onchocerciasis was a country-wide public health problem with an estimated 12 million people at risk [9,10]. The Ministry of Health (MoH) started the community-directed treatment with ivermectin (CDTI) strategy in the West Region in 1996, initially with the support of The Carter Center, Lions Clubs International Foundation (LCIF) and the African Programme for Onchocerciasis Control (APOC) and then Sightsavers [11,12].

Impact evaluations conducted in the region in 2011, found that only 3 out of 11 health districts (HDs) were close to the elimination targets despite 16 years of uninterrupted MDA [12]. In particular, there were still very high levels of infection (humans and flies) in selected sentinel sites in Massangam and Foumbot HDs. In Massangam, 0.18% of flies were infective and 59.6% of individuals had mf, whilst in Foumbot, 0.19% of flies were infective and 41.9% and 12.7% of individuals had mf from the two sentinel sites [12]. In 2014, a study by Sightsavers in collaboration with the MoH investigated the potential local and micro-level determinants that could be facilitating on-going transmission in these two HDs, including poor ivermectin coverage [11]. In recent years, the MoH have reported high annual ivermectin coverage including over 80% therapeutic and 100% geographic coverage across Massangam district since 2007. Senyonjo *et al*. aimed to validate the 2014 ivermectin coverage reported by community drug distributors (CDDs) through an independent treatment coverage survey and understand barriers to compliance using a mixed methods approach. The authors concluded compliance to ivermectin was relatively good (71.2%, 95%CI: 61.7–79.2%) although a little under the 80% coverage target as set by WHO. However, compliance was an issue in certain population groups due to both programme delivery issues and individual determinants, which need to be addressed. With this in mind, the authors concluded that due to very high infection rates, especially in the sentinel community of Makouopsap, Massangam HD, that improving compliance alone would not be enough to interrupt transmission [11]. A change in intervention strategy would be required to achieve elimination by 2025.

In order to inform strategic programmatic changes, further studies were required to better understand the transmission dynamics in the area. The study described in this paper aimed to delineate the boundaries of the area of high transmission, firstly through mapping of the breeding sites of *S*. *damnosum* and assessment of key entomological and parasitological parameters at purposefully selected sites, within the flight range of the vector from the breeding sites identified. Parasitological and entomological surveys were conducted in 2015 and 2016. It should be noted that the study was not aiming to delineate the wider transmission zone but understand the boundaries of any area of high transmission within the zone, in order to inform changes in programmatic interventions.

## Methods

### Ethics statement

This study was approved by the Comité National d’éthique de la Recherche pour la Santé Humaine in Cameroon (approval number 2014/12/519/CE/CNERSH/SP). The administrative authorization to collect the data was obtained from the MoH (approval number 631–3715). The aim and objectives of the studies were explained to all potential participants or caregivers for those aged less than 21 years old and written informed consent was obtained and recorded. All operators of the aspirators were covered to minimise skin exposure to *Simulium* bites.

### Study area

The West Region has an estimated population of about 1.7 million people living across 20 HDs. It is among the most mountainous zones of the country with peaks reaching up to 3000 metres above sea level. These physical features create many perennial fast-running rivers that support breeding of black flies throughout the year. In the west and east of the region there are thick forests, whilst the central area transitions from a forest to savannah woodland [12]. The study was conducted in the Massangam HD in the West Region of Cameroon.

Massangam HD, with a population of approximately 40,000 [11], is situated in the extreme east of the region. There are two main rivers in the area, the Noun and the Mbam rivers which lie to the south-west and the east of Massangam respectively. The HD is a watershed from which many tributaries of the River Mbam flow, the main one being the River Nja and a second the River Kim.

The heavy rainy season lasts from August to November and the dry season from December to March. This is then followed by a small rainy season from April to June and a short dry season in July.

### Study design

The study used a variety of methods, which included both parasitological cross-sectional studies and entomological surveys. Figure maps were created using ArcGIS software (ESRI 2011. ArcGIS Desktop: Release 10. Redlands, CA: Environmental Systems Research Institute).

### Entomological study

#### Prospecting for *S*. *damnosum* breeding sites

**Fig 1:**
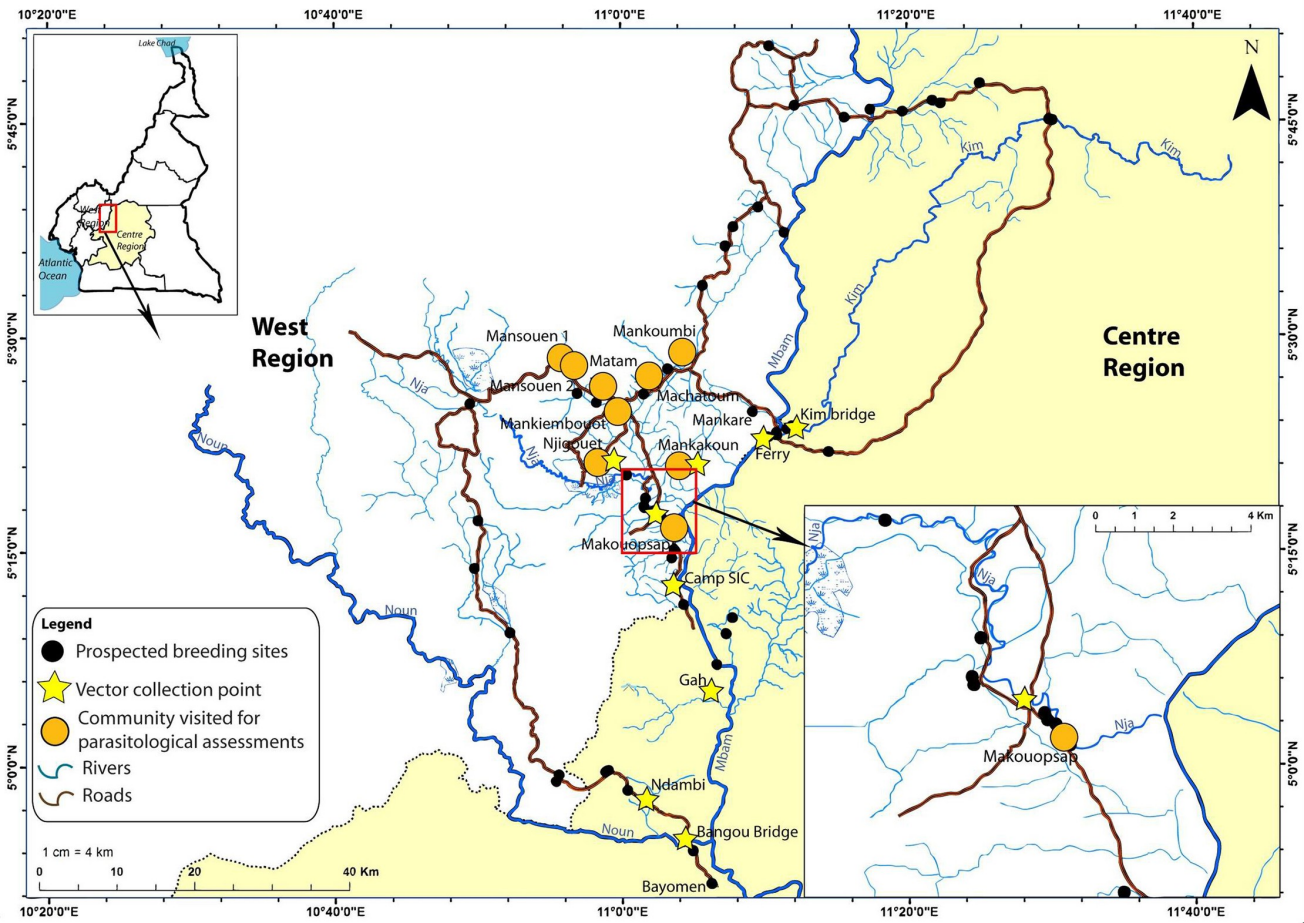
Map of the communities where parasitological assessments were conducted, locations of prospected sites for *S*. *damnsoum* larvae and vector collection points. Fig 1 was adapted from open source maps retrieved on Diva-GIS at www.diva-gis.org/gdata and on USGS at https://www.usgs.gov/products/maps/topo-maps.

Potential breeding sites in the main rivers and tributaries in the study area (within the approximate flight range of *S*. *damnosum* from Makouopsap sentinel community) were visited and prospected for *S*. *damnosum* larvae, (Fig 1).

The initial prospection was conducted in the dry season (July 2015) but a second prospection was carried out in the rainy season (April 2016) in order to visit additional potential breeding sites that were not accessible during the initial prospection. The search was also further extended to include the River Kim (an important tributary of the Mbam River in North-West Massangam) and further potential breeding sites on the River Mbam, in the neighbouring Bafia HD, Central Region.

Any mature larvae (6 and 7 stages) found were preserved in Carnoy fixative and labelled (date, river name, number) for cytotaxonomic studies at Noguchi Memorial Institute for Medical Research in Ghana. All breeding sites were identified by their coordinates using a portable geographical positioning system (GPS) device.

#### Collection, identification and dissection of adult *S*. *damnosum* females

Adults of *S*. *damnosum* were collected at vector collection sites selected near identified or suspected breeding sites or communities of interest. Two collections were carried out, the first one during the dry season (9 days) in July 2015 and the second one during the rainy season (up to 7 days) in April 2016. These two seasons influence *S*. *damnosum* activity differently and it was important to conduct vector collection activities across the two periods. In the dry season, two black fly collection sites were chosen, one by the banks of the Mbam (Camp Sic) and one in the sentinel community of Makouopsap. In the rainy season nine sites were selected for fly collection, the two used in the dry season and in an additional seven communities and locations along the River Mbam, River Nja, River Kim and River Noun (Fig 1 and Table 1).

**Table 1:** Collection and dissection of *S*. *damnsoum* caught during the rainy season.

River	Catching point	# of days	# caught	# dissected	% of dissected flies parous (# parous)	% of parous flies infected (# infected)	% of parous flies infective (# infective)	# L3H (in all infective flies)	L3H/ infective fly
Mbam	Camp SIC	7	6,683	2,047	30.0 (616)	0.8 (5)	0.6 (4)	12	3
	Mankakoun	4	699	436	36.0 (157)	0.6 (1)	0.6 (1)	1	1
	Ferry near Mankare	1	620	305	39.3 (120)	1.7 (2)	0.8 (1)	3	3
	Gah	2	2,419	1,112	22.3 (248)	1.2 (3)	0.4 (1)	1	1
Nja	Makouopsap **	7	2,465	1,259	25.9 (326)	1.2 (4)	0.9 (3)	8	2.7
	Njingouet	2	20	20	0	0	0	0	0
Kim	Kim bridge near Manakre	2	303	103	48.5 (50)	2.0 (1)	2.0 (1)	3	3
Noun	Bangou (bridge)	2	1,678	434	50.0 (217)	0.9 (2)	1.4 (3)	7	2.3
	Ndambi	1	353	251	21.9 (55)	0	0	0	0
**Total**		** **		**15,240**	**5,967**	**30.0 (1,789)**	**1.0 (18)**	**0.8 (14)**	**35**	**2.5**

** In 2011, 0.18% of flies were infective [12]

Black flies were collected using modified Esperanza Window Traps and battery-run insect aspirators. This trap was originally designed by Rodriguez-Perez and colleagues [13] for use in Latin America to collect *Simulium ochraceum* complex and has been optimized and adapted to collect African *S*. *damnosum* flies, details of which have been described by Toe *et al* [14]. In summary, the original black satin cloth was replaced by a deep blue equivalent, keeping the original dimensions. The trap was placed inside a wooden frame and the lower end raised from the ground by two wooden pieces of 60cm high. To attract the *S*. *damnosum*, two materials were used, CO_2_ generated by dissolving 17.5g of Baker’s yeast and 250g of sugar in 2.5 litres of water [15,16] and worn trousers/shirts. The commercial skin-lure (BG-Lure attractant, Biogents AG, Regensburg, Germany) could not be obtained for this study. In the dry season collection, a suitable glue for the trap was not available and therefore instead of the flies being immobilised by the glue, they were sucked up using the aspirators.

For the collection of flies in the rainy season the modified Esperanza Window Trap was also used. Tangle Trap ^TM^ insect trap coating paste (Contech, Victoria, BC, Canada) and Tanglefoot ^TM^, (Grand Rapids, Michigan, USA) were used to immobilize the insects that landed on the traps. In addition, human bait was also used, with one individual at each collection point wearing a cylindrical shaped satin material (black and blue), flies were collected by another individual as they landed using a battery-run insect aspirator. Flies were collected from 8 am to 4 pm. The number of days that flies were collected differed from one to seven days per site, the days are outlined in Table 1.

Black flies were identified morphologically and were dissected to assess the infection rates during the respective periods. The main morphological characters used for the identification were: the color of the scutellum, wing tufts and arculus of the antennae, the procoxa and the 9^th^ abdominal tergit [17].

#### Parasitological studies

Parasitological studies were conducted in October 2015, a year since the last round of ivermectin MDA conducted in October 2014. A total of 10 communities were visited for the parasitological assessment (Fig 1), selected based on their proximity to identified *S*. *damnosum* breeding sites.

#### Registration of study participants

Each household in the selected communities were visited for the study (exhaustive sampling). Individuals residing in the households were eligible for inclusion if they were aged at least three years old and had lived in the community since birth or for a minimum of five years. Informed consent was sought from all participants (or their caregivers for minors) and basic demographic information (name, age, sex, length of stay in the community) was recorded. Each individual was tracked using a unique identifier code. Clinical examinations were conducted at a central location.

#### Nodule palpation

Nodule palpations were conducted in a well-lit but private setting. The torso was examined for nodules starting with the head down to the neck, the chest, the iliac crest, the trochanter, the legs (especially the knees) and the feet. Close attention was paid to the bony protuberances. Any nodules found were recorded (number, position). Nodule palpation is no longer recommended as a key indicator to assess progress towards onchocerciasis elimination [18] because it has poor specificity and sensitivity for *O*. *volvulus* infection, especially in low prevalence settings. However, we included it here to use as a comparison to baseline data where the indicator was collected.

#### Skin snip procedure

Two bloodless skin snips (one from each iliac crest) were taken from each individual using a sterile 2mm Holtz corneo-scleral punch. The skin snip was then placed into a well in which two drops of saline solution had been previously placed. When all the wells on the micro-titer plate had been filled, the plate was sealed with parafilm to avoid spillage and evaporation. The skins were incubated for 24 hours at room temperature. Punches were placed in an alcohol bath for five minutes, transferred to a hypochlorous acid (Eau de Javel or Chlorox) bath and then rinsed in distilled water, before sterilizing in a pressure cooker for at least one hour. After 24 hours, the incubation medium was examined for the presence or absence of any mfs using a compound microscope (x10). After examination, the skin snips were fixed with two drops of 80% alcohol [12,19]. Initially all individuals were targeted for skin snip procedure but due to resistance in the community, this was later revised and only adults (aged above 15 years) were offered a biopsy.

#### Rapid immunochromatographic test (Ov-16)

The onchocerciasis Ov-16 immunochromatographic assay (Bio Line SD Onchocerciasis IgG_4_ of Standard Diagnostics Inc.) was used to detect antibodies against Ov-16 antigen. All children aged 3 to 10 years old residing in the 10 communities were eligible and tested if present (exhaustive sampling). WHO recommends the detection of anti Ov-16 antibodies in children aged less than 10 years. This study also included those aged 10 years in order to better understand the transmission dynamics in this age group but they are reported separately to keep in line with WHO reporting requirements [20]. The participant’s finger was pricked using a sterile one-use lancet and 10μl of blood was placed in the sample well of the test strip. Four drops of buffer where then added to the chase buffer well. Tests were read after 20 minutes as specified in the test protocol [21]. Any tests that had no control line were re-done.

#### Data analysis

All data was entered into Excel and analysed using the statistical package STATA, version 13.0 (TX: StataCorp LP).

The community microfilarial load (CMFL) was calculated as the geometric mean of the number of microfilariae per skin snip in adults aged 20 years and above. As this measure includes those with a mf count of zero, the mean was calculated after a log(x + 1) transformation, where x is the individual microfilarial density.

The non-parametric test for trend was used to determine an association between Ov-16 seroprevalence and increasing age. Chi-square tests were used for other univariate tests of association between the response variables (Ov-16 seroprevalence, mf prevalence and nodule prevalence) and sex.

Missing values were excluded from the analysis.

## Results

### Breeding sites

**Fig 2:**
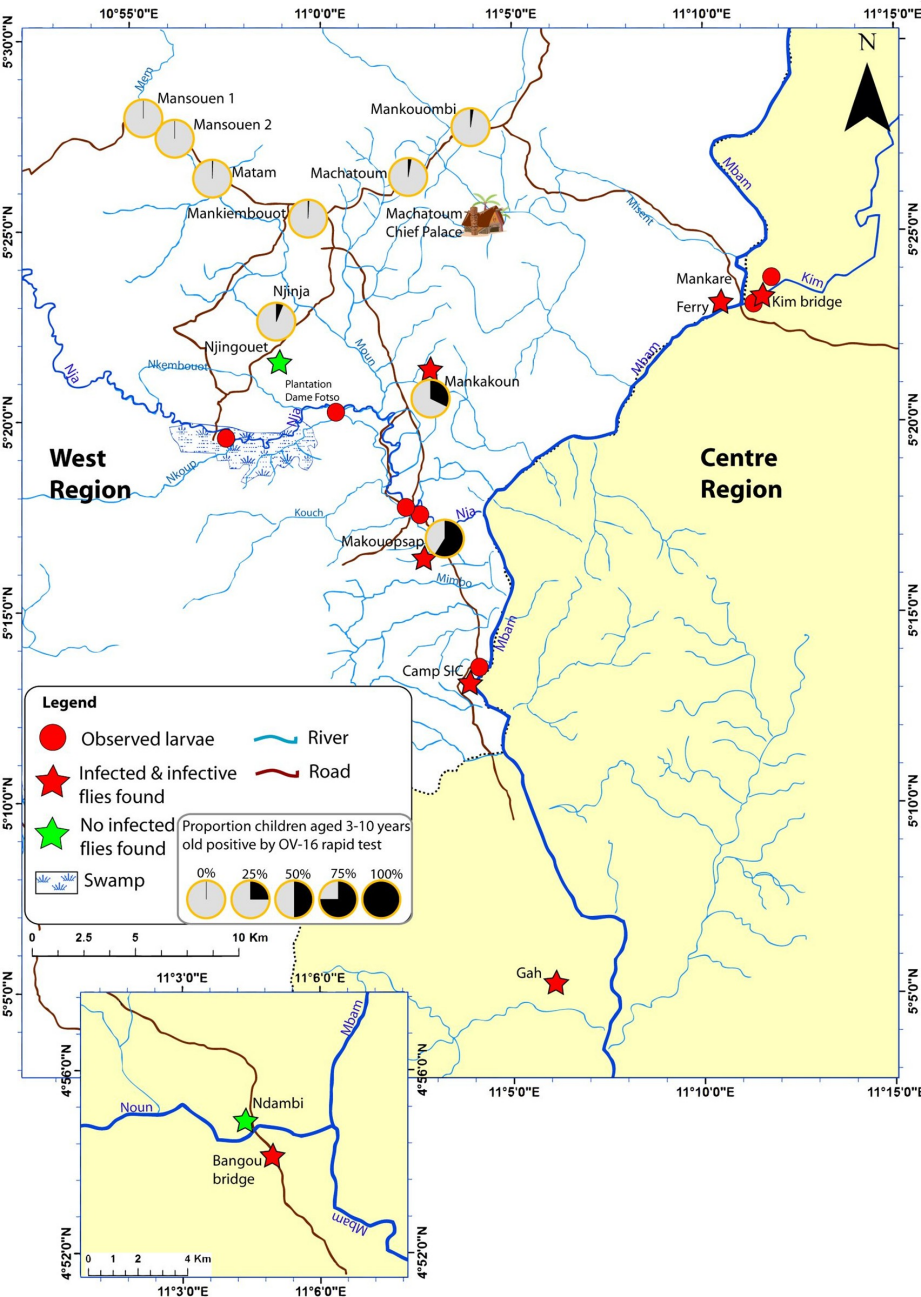
Identified breeding sites, locations where infective and infected black flies were caught and results of Ov-16 seroprevalence in children. Fig 2 was adapted from open source maps retrieved on Diva-GIS at www.diva-gis.org/gdata and on USGS at https://www.usgs.gov/products/maps/topo-maps.

A total of seven breeding sites were identified in the study area, these included one on the River Mbam, four on the River Nja and two on the River Kim (Fig 2). Two additional breeding sites were identified on the River Noun but due to their distance from the sentinel community, it is believed they are unlikely to support transmission in this area.

Of the three main rapids of interest on the River Mbam, only one was found to have *S*. *damnosum* larvae. No larvae were found in the potential breeding site to the north of the study area, however larvae were found in one of the two rapids to the south. Due to the volume of water these sites could not be re-prospected during the rainy season.

Of the eight rapids on the River Nja, three were productive in the dry season (two in close proximity to the sentinel community of Makouopsap) and one a distance upstream, an additional breeding site was also identified upstream (from Makouopsap) in the rainy season.

No further *S*. *damnosum* larvae were found in any of the other tributaries prospected.

### Fly catches

In the dry season, a total of 4,598 female *S*. *damnosum* were collected from the two different catching points, over a nine day period in July. Out of this number, 3,162 were dissected and 20.0% (633) were found to be parous. A total of 1.3% of parous flies were infected (n = 8, 0.3% of all flies) while 0.3% (n = 2) were infective, each of them having two infective larvae in the head. The majority (95%) of the black flies were caught from the site near the River Mbam.

During the rainy season, a total 15,240 flies were collected from nine different sites. Using landing rates as a proxy for biting rates, the highest rates were at Gah with 1,209 flies/day and on the shores of the River Mbam (Camp SIC) with a total of 955 flies/day. Makouopsap (sentinel community) had a landing rate of 352 flies/day.

A total of 30% (range 0–50%) of all flies were parous, with the highest proportion of parous flies from the collection points near the Rivers Kim and the Noun. In all, 1.0% of all parous flies were found to be infected and 0.8% infective. Again the highest proportion of infective flies were at the catching points by the Rivers Kim and Noun, however, there was also a significant proportion of infective flies identified in Makouopsap. Overall, there was about 2.5 L3H (third stage infective larva found in the head) per infective fly (23 L3H/1000 parous females), with the highest proportion in Makouopsap (Table 1).

Using morphological characteristics, it was concluded that the vector for onchocerciasis in the area belongs to the forest cytospecies and could be S. *squamosum sensu stricto (ss)* (a member of the *S*.*squamosum* sub complex) as previously identified from the area by D. Boakye.

### Parasitological findings

#### Clinical examinations

In total 2,377 individuals from 10 different communities participated in the study (although Njingouet and Njinja are considered one community for the purposes of this study). The median age of participants was nine years old. The nodule rates were highest in Makouopsap community (40.0% in adults over 15) followed by Mankakoun (34.2% in adults aged over 15). Nodules were identified in young children in both of these communities. At least one person with nodules were identified in all communities (Table 2).

**Table 2:** Prevalence of nodules per age group in 10 communities in the Massangam HD, October 2015.

Communities	3–9 years old		10–15 years		> 15 years		Total	
	#	% positive (#)	#	% positive (#)	#	% positive (#)	#	% positive (#)
Makouopsap**	79	6.3 (5)	20	35.3 (6)	35	40.0 (14)	131	19.1 (25)
Machatoum	220	0	17	0	124	7.2 (9)	361	2.5 (9)
Mankakoun	145	3.4 (5)	53	3.8 (2)	38	34.2 (13)	236	8.5 (20)
Mankiembouot	94	0	23	0	57	1.8 (1)	174	0.6 (1)
Mankoumbi	183	0	48	4.2 (2)	134	5.2 (7)	365	2.5 (9)
Mansouen 1	40	0	40	0	82	9.8 (8)	162	8 (4.9)
Mansouen 2	112	0	41	0	91	4.4 (4)	244	4(1.6)
Matam	314	0	131	0	69	1.5 (1)	514	1 (0.2)
Njingouet/Njinja	121	0	18	5.6 (1)	51	11.8 (6)	190	7(3.7)
** Total**	**1,308**	**0.8 (10)**	**388**	**2.8 (11)**	**681**	**9.3 (63)**	**2,377**	**3.5 (84)**

** data from the sentinel site in 2011 reported 43.4% crude nodule prevalence in adults [12]

Of those with nodules, the average number of nodules per person was 1.4, the highest number identified on one individual was five, a male aged 50 years old. There was evidence of an association between presence of nodules and sex, with 4.7% of males with nodules as compared to 2.3% of females (p = 0.002).

#### Parasitological examinations

In total, 898 individuals had a skin snip taken, with a median age of 30 years old. Initially children were offered a skin snip biopsy but there were a high rate of refusals, so the study team concentrated on offering a skin snip to individuals aged above 15 years old.

A total of 78 skin snips taken from adults aged over 15 years were positive (11.5%). The highest rate was recorded in Makouopsap (37.1%) followed by Mankakoun (36.8%) and Njigouet/Njinja (27.5%) (Table 3). There was significant infection identified across all age groups in these communities. There was no association between age and presence of mf in adults aged above 15 years (z score = -0.5; p = 0.62). There was a strong association between prevalence of microfilaria and sex, with 17.4% of males having mf as compared to 9.0% in females (p<0.001).

**Table 3:** Prevalence of microfilariae (mf) and community microfilaria load (CMFL) in 10 communities in the Massangam HD, Oct 2015.

Communities	#	% positive in adults aged >15 years (#)	CMFL (mf/skin snip)
Makouopsap**	35	37.1 (13)	0.57
Machatoum	124	8.1 (10)	0.13
Mankakoun	38	36.8 (14)	0.89
Mankiembouot	56	5.4 (3)	0.08
Mankoumbi	134	9.7 (13)	0.13
Mansouen 1	82	8.5 (7)	0.11
Mansouen 2	91	1.1 (1)	0.01
Matam	69	4.3 (3)	0.08
Njingouet/Njinja	51	27.5 (14)	0.56
**Total**	**680**	**11.5 (78)**	**0.18**

In Makouopsap, a total of 39 children under the age of 16 were tested for mf (before the protocol changed). A total of 51.3% were mf positive, 57.7% of those aged 3–9 years old (n = 26) were mf positive. In Mankoukoun, a total of 23 children under the age of 16 were tested for mf, of which 43.5% were mf positive.

The overall CMFL, an indicator of the intensity of infection in a community, is 0.18 mf per skin snip. The highest CMFL was recorded in Mankakaoun (0.89 mf/skin snip) and Makouopsap (0.57mf/skin snip), (Table 3). The most heavily infected person was from Mankoumbi with an average of 141.5 mf per skin snip.

#### Ov-16 seroprevalence

A total of 145 out of 1,518 (9.6%) children aged 3 to 9 years old tested had antibodies to Ov-16. Makouopsap community had the highest seroprevalence (59.0%) followed by Mankakoun (32.4%) and Njigouet/Njinja (6.2%). In comparison, a number of communities further in distance from the sentinel community of Makouopsap and productive breeding sites on the River Nja, had no seropositive children (Table 4). This suggests an area of high transmission constituting of the four communities of Makouopsap, Mankakoun and Niigouet/Njinja (Fig 2).

**Table 4:** Ov-16 seroprevalence per age group in 10 communities in the Massangam HD.

Communities	3–6 years old		7–9 years old		Total (3 to 10 years)	
	n	% (n positive)	n	% (n positive)	n	% (n positive)
Makouopsap	35	28.6 (10)	36	83.7 (36)	85	59.0 (46)
Machatoum	125	1.6 (2)	93	4.3 (4)	218	2.8 (6)
Mankakoun	71	22.5 (16)	74	41.9 (31)	145	32.4 (47)
Mankiembouot	41	2.4 (1)	53	0	94	1.1 (1)
Mankoumbi	131	1.5 (2)	52	5.8 (3)	183	2.7 (5)
Mansouen 1	15	0	25	0	40	0
Mansouen 2	76	0	36	0	112	0
Matam	134	0	180	1.1 (2)	314	0.6 (2)
Njingouet/Njinja	83	2.4 (2)	46	13.0 (6)	129	6.2 (8)
**Total**	**711**	**4.6 (33)**	**807**	**13.9 (112)**	**1,518**	**9.6 (145)**

There was very weak evidence of a difference in seropositivity by sex, with seropositivity slightly higher in males (9.9%) as compared to females (7.2%), (p = 0.08). There was a strong association between increasing age and seroprevalence (z = 6.29; p<0.001).

## Discussion

The parasitological and entomological findings suggest there is an area of perennial high transmission in Massangam HD, encompassing four communities of Makouopsap, Mankakoun and Njingouet/Njinja and facilitated by *S*. *damnosum* breeding sites on the rivers Nja and Mbam.

The entomological assessments showed that infective flies were identified throughout both the dry and rainy seasons, with the sites being more productive during the rainy season. Similar results were reported by Katabarwa and colleagues who showed an increase in biting rates in Makouopsap from January to June [12]. In the rainy season, a total of 0.8% of parous flies were infective, higher than the figures (0.18%) reported by Katabarwa and colleagues [12] and higher than elimination thresholds of 0.1% [20].

Prospection of the River Mbam, especially in the rainy season was restricted by accessibility issues and high water levels, compounded by a recent release of volumes of water from the two retention dams (Magba and Bamendjing) to regulate hydroelectricity supplies. However, the vector collection sites chosen near the River Mbam in the rainy season were very productive and the flies had relatively high rates of infection, especially at the site near Camp SIC and downstream at Gah (highest biting rate per day recorded). The large number of flies caught at sites along the river shores even in the dry season, imply the River Mbam is a main perennial source of biting *S*. *damnosum* especially between Mankare and Makouopsap down to Gah (about 30km south of the sentinel community, Makouopsap). Within this section of the Mbam there are several rapids, of which *S*. *damnosum* larvae were positively identified in one, although suspected in the other rapids due to high vector landing rates in near-by catching points. Even when the water levels are low, *Simulium* flies can still find isolated substrates in the rapids to enable them to reproduce.

The parasitological assessments confirm the sentinel community of Makouopsap is still endemic, despite 20 years of treatment with ivermectin. Overall, nodule and mf prevalence have decreased since the 2011 study by Katabarwa *et al* that reported 43.4% of individuals over 20 years had nodules and 59.6% mf as compared to 19.1% (40% in adults) and 37.1% in this study. Another study by Kamga *et al* conducted in Makouopsap in May 2015, reported a slightly higher CMFL of 0.9 (mf/ss) in Makouopsap as compared to 0.57 (mf/ss) in this study [22]. These are positive findings suggesting some progress is being made in reducing *O*. *volvulus* prevalence, intensity and transmission but that the study area is still off track to reach current elimination targets.

There was a high prevalence of antibodies to Ov-16 in children in the sentinel community with 59.0% testing positive. The neighbouring communities of Mankakoun (32.4%) and Njingouet/Njinja (6.2%) also have high seropositivity rates in children, well above the WHO elimination threshold (0.1%) [20]. In fact, only two of the nine communities in the study reached the elimination criteria, although the seropositivity rates in the remaining communities were all below 3%. Although the Ov-16 assay is only a measure of exposure to *O*. *volvulus*, as it was conducted in children born after MDA began and positive cases were found in very young children, this does provide evidence of recent and on-going transmission especially around the sentinel community of Makouopsap and the neighbouring breeding sites on the River Nja. A predominant source of income in these communities is farming and many adults take their children with them to the fields, especially during the holidays and infection in children in these communities could be a result of these practices.

The data from the remaining sampled communities (outside this area of high transmission) suggest they are closer to reaching elimination thresholds. The communities of Mansouen 1 and 2, Matam and Mankimbouot (Fig 2) likely have no *in situ* transmission with no or very low levels of antibodies detected in children. Machatoum and Mankoumbi, which are nearer the River Mbam may experience brief periods of invasion by migrant black flies during peak biting season and therefore some seasonal transmission may be occurring.

The higher levels of infection in males is interesting and similar associations have been reported elsewhere [23]. The difference is potentially a result of gender roles which put males at more risk of black fly biting [24], such as fishing practices or herdsmanship known to be important livelihood practices in this area. Although differences in gender and compliance to ivermectin could also be a factor, previous research in this area found no difference in overall ivermectin compliance after the 2014 MDA round by sex. In fact, the authors further concluded that systematic non-compliers (never taken the drug) were more likely to be female and this was linked to the fears that ivermectin could affect fertility or interrupt menstruation [11]. A study by Kamga *et al* (2017) also found little evidence between the geometric mean of mf among carriers and treatment adherence in the West Region of Cameroon [22].

A further consideration is the potential source of black flies flying in from the south from the productive breeding sites on the River Noun. It is also an area where flies may be feeding on the cattle that were observed transiting through this area as they move north. However, the distance between the identified breeding sites on the River Noun and the sentinel community of Makouopsap, approximately 40km, suggests that these breeding sites may have little influence on the area of high transmission in Massangam HD.

There are a number of methodological issues that need to be taken into account when interpreting results of this study and in future similar research. First, the entomological evidence suggests that there is on-going transmission of *O*. *volvulus* in Massangam HD. However, PCR using *O*.*volvulus* specific PCR primers were not employed and therefore there is a possibility that some of the parasites found in the flies could be *O*. *ochengi*, as found elsewhere in Cameroon [25], especially as there are substantial population of cattle in the area, the reservoir for this species of *Onchocerca*. Current WHO guidelines only recognize the use of PCR and species-specific primers in order to determine *O*. *volvulus* infection rates in flies [18]. However, although PCR was not used in this study, opening up the possibility of misinterpretation of the true infection levels in flies, the high proportion of individuals infected with *O*. *volvulus* do suggest that the majority of *S*. *damnosum* are truly infected with *O*. *volvulus*. The study would be strengthened if flies could have been collected over a longer period of time, to collect a larger number of flies and to be able to determine monthly and annual biting rates.

There are also difficulties in using morphological characteristics only to identify the exact species of adult *S*. *damnosum*. However, based on previous cytotaxonomic identification of larvae collected in these rivers, the suggestion is that the species are likely forest flies belonging to *S*. *squamosum*. It would be preferable to confirm the species and infectivity using DNA analysis, although there are no validated molecular tests for species identification of *S*. *damnosum* complexes at present.

Further, a combination of modified Esperanza traps and human bait collections were used. During this study we were not able to obtain the commercial skin-lure (BG-Lure attractant, Biogents AG, Regensburg, Germany). In the dry season it was not possible for us to buy the Tangle TrapTM insect trap coating paste (Contech, Victoria, BC, Canada) and although another type of glue (TanglefootTM, Grand Rapids, Michigan, USA) was tried, it acted as a repellent. The lack of standardisation of traps and human collections used makes it more difficult to compare data across time periods.

There are issues with using nodule prevalence as a parameter of infection, due to the poor specificity of the indicator [26], although the combination of parasitological data collected during this study confirms that onchocerciasis is still prevalent in Massangam district. There were issues of high rates of refusal for skin snip biopsies in children in the study communities, which resulted in a change of inclusion criteria after the start of the data collection. The low response rate in children for skin snip biopsies were offset by the higher response rate in the use of the Ov-16 assay, which was more useful in determining historical transmission dynamics in the area. The skin snips were also not weighed, which would have provided a better indication of CMFL, although efforts were made to standardize the skin biopsy taken so the variability in weight should not have been large.

To conclude, both parasitological and entomological findings confirm an ongoing transmission of *O*. *volvulus* in the Massangam HD despite 20 years of annual mass distribution of ivermectin. The study was able to refine the boundaries of an area of high perennial transmission (approximately 12km) within the wider transmission zone, which was around the sentinel site of Makouopsap and facilitated by productive breeding sites on the River Nja and River Mbam.

The evidence from past studies suggests that this area of high transmission is not a failure to treat ie a result of poor coverage or compliance [11] but is more likely a failure of the treatment strategy. Although ivermectin is effective as a microfilaricide, recuperation of fertility and repopulation of the skin with mf begins three to four months after the drug is ingested [27]. In an area of perennial transmission such as identified in Massangam HD, this mf repopulation in the skin, combined with the abundance of vectors to pick and transmit the *O*. *volvulus* parasite, indicate annual ivermectin alone is likely not enough to interrupt transmission. Alternative strategies such as semi-annual ivermectin delivery or use of a more effective microfilaricide such as moxidectin could be beneficial in ensuring suppression of mf levels in the population for longer time periods [28]. However, as there is evidence of recent transmission in the area and the lack of a macrofilaricidal effect of these drugs, even with the introduction of these interventions, 2025 elimination targets would unlikely be met.

We therefore advocate for a multi-faceted approach aimed at targeting the remaining significant reservoir of infection. Such an alternative intervention would comprise of a test and treat strategy with a known macrofilaricide such as doxycycline, targeting communities in the area of high transmission, combined with associated ground larviciding of relevant *S*. *damnosum* breeding sites. Doxycycline is known to target the *O*. *volvulus Wolbachia* endosymbiont resulting in the increased sterility and death of the adult worm [29]. Ground larviciding is a complementary approach that will target the life cycle of the black fly vector and an approach used to great success in West Africa during the Onchocerciasis Control Programme (OCP) days [30,31]. In an effort to ensure low mf prevalence in communities surrounding this area of high transmission and help reduce the potential of re-introduction of infection into the area, we also suggest increasing ivermectin distribution from annual to semi-annual delivery. Sustaining high treatment coverage will be very important and it will be necessary to tailor efforts to improve ivermectin compliance, especially in certain groups that consistently do not take the drug e.g nomadic groups that travel in and out of the area. Interventions should also be co-ordinated with on-going elimination efforts in the Central Region that have very high force of infection and neighbour Massangam district [12].

## Supporting information

S1 Table STROBE checklist. (DOCX)
