# Supplementary material for: On-going transmission of human onchocerciasis in the Massangam health district in the West Region of Cameroon: Better understanding transmission dynamics to inform changes in programmatic interventions
Source: PLoS Negl Trop Dis. 2018 Nov 14;12(11):e0006904. doi: 10.1371/journal.pntd.0006904 (PMC6261645; doi:10.1371/journal.pntd.0006904)
Supplement: S1 Table — (DOCX) [file pntd.0006904.s001.docx]

STROBE Statement—checklist of items that should be included in reports of observational studies

|  | Item No. | Recommendation | Page  No. | Relevant text from manuscript |
| --- | --- | --- | --- | --- |
| **Title and abstract** | 1 | (*a*) Indicate the study’s design with a commonly used term in the title or the abstract | 2 | Parasitological, entomological surveys, prospection of potential breeding sites |
|  |  | (*b*) Provide in the abstract an informative and balanced summary of what was done and what was found | 2 |  |
| Introduction | | | |  |
| Background/rationale | 2 | Explain the scientific background and rationale for the investigation being reported | 4-5 | Impact evaluations conducted in the region in 2011, found that only 3 out of 11 health districts (HDs) were close to the elimination targets despite 16 years of uninterrupted MDA (12). In particular, there were still very high levels of infection (humans and flies) in selected sentinel sites in Massangam and Foumbot HDs.  In order to inform strategic programmatic changes, further studies were required to better understand the transmission dynamics in the area. |
| Objectives | 3 | State specific objectives, including any prespecified hypotheses | 5 | The study described in this paper aimed to delineate the boundaries of the focus of high transmission around Makouopsap sentinel village through mapping of the nearby breeding sites of *S. damnsoum* and assessment of key entomological and parasitological parameters at purposefully selected sites, specified distances from the sentinel village. |
| Methods | | | |  |
| Study design | 4 | Present key elements of study design early in the paper | 5-9 | The study used a mixed method design, which included both parasitological cross sectional studies and entomological surveys. Further details given |
| Setting | 5 | Describe the setting, locations, and relevant dates, including periods of recruitment, exposure, follow-up, and data collection | 5  6-9 | Overview of area given  Data collection dates |
| Participants | 6 | (*a*) *Cohort study*—Give the eligibility criteria, and the sources and methods of selection of participants. Describe methods of follow-up  *Case-control study*—Give the eligibility criteria, and the sources and methods of case ascertainment and control selection. Give the rationale for the choice of cases and controls  *Cross-sectional study*—Give the eligibility criteria, and the sources and methods of selection of participants | 6  7  8 | Potential breeding sites in the main rivers and tributaries in the study area (within the approximate flight range of S. damnosum from Makouopsap sentinel village) were visited and prospected for S. damnosum larvae,  A total of 10 communities were visited for the parasitological assessment, figure 1, selected based on their proximity to the sentinel village (Makouopsap) and identified S. damnosum breeding sites.  Each household in the selected communities were visited for the study (exhaustive sampling). Individuals residing in the households were eligible for inclusion if they were aged at least three years old and had lived in the community since birth or for a minimum of five years. |
|  |  | (*b*) *Cohort study*—For matched studies, give matching criteria and number of exposed and unexposed  *Case-control study*—For matched studies, give matching criteria and the number of controls per case | NA |  |
| Variables | 7 | Clearly define all outcomes, exposures, predictors, potential confounders, and effect modifiers. Give diagnostic criteria, if applicable | 6  8-9 | Potential breeding sites in the main rivers and tributaries in the study area (within the approximate flight range of S. damnosum from Makouopsap sentinel village) were visited and prospected for S. damnosum larvae. Any mature larvae (6 and 7 stages) found were preserved in Carnoy fixative and labelled (date, river name, number) for cytotaxonomic studies.  Adults of S. damnosum were collected at vector collection sites selected near identified or suspected breeding sites or villages of interest  Description of nodule palpation, skin snip biopsy and ov-16 test given |
| Data sources/ measurement | 8* | For each variable of interest, give sources of data and details of methods of assessment (measurement). Describe comparability of assessment methods if there is more than one group | 6-9 | Details as above |
| Bias | 9 | Describe any efforts to address potential sources of bias |  |  |
| Study size | 10 | Explain how the study size was arrived at | 8 & 9 | Exhaustive sampling was used  Sites for sampling were selected based on distance from the sentinel site of Makouopsap |

Continued on next page

| Quantitative variables | 11 | Explain how quantitative variables were handled in the analyses. If applicable, describe which groupings were chosen and why | 10 | Explanations of analysis given |
| --- | --- | --- | --- | --- |
| Statistical methods | 12 | (*a*) Describe all statistical methods, including those used to control for confounding | 10 | The community microfilarial load (CMFL) was calculated as the geometric mean of the number of microfilariae per skin snip in adults aged 20 years and above. As this measure includes those with a mf count of zero, the mean was calculated after a log(n + 1) transformation, where n is the number of mf per snip. |
|  |  | (*b*) Describe any methods used to examine subgroups and interactions | 10 | The non-parametric test for trend was used to determine an association between Ov-16 seroprevalence and increasing age. Chi-square tests were used for other univariate tests of association between the response variables (Ov-16 seroprevalence, mf prevalence and nodule prevalence) and sex. |
|  |  | (*c*) Explain how missing data were addressed | 10 | Missing values were excluded from analysis |
|  |  | (*d*) *Cohort study*—If applicable, explain how loss to follow-up was addressed  *Case-control study*—If applicable, explain how matching of cases and controls was addressed  *Cross-sectional study*—If applicable, describe analytical methods taking account of sampling strategy | NA |  |
|  |  | (*e*) Describe any sensitivity analyses | NA | None conducted |
| Results | | | | |
| Participants | 13* | (a) Report numbers of individuals at each stage of study—eg numbers potentially eligible, examined for eligibility, confirmed eligible, included in the study, completing follow-up, and analysed | 13, 15 |  |
|  |  | (b) Give reasons for non-participation at each stage | 14 | Initially children were offered a skin snip biopsy but there were a high rate of refusals, so the study team concentrated on offering a skin snip to individuals aged above 15 years old. |
|  |  | (c) Consider use of a flow diagram |  | Not required |
| Descriptive data | 14* | (a) Give characteristics of study participants (eg demographic, clinical, social) and information on exposures and potential confounders | 13, 14 | Median age |
|  |  | (b) Indicate number of participants with missing data for each variable of interest | 14-15 | Data available in tables |
|  |  | (c) *Cohort study*—Summarise follow-up time (eg, average and total amount) | NA |  |
| Outcome data | 15* | *Cohort study*—Report numbers of outcome events or summary measures over time | *NA* |  |
|  |  | *Case-control study—*Report numbers in each exposure category, or summary measures of exposure | *NA* |  |
|  |  | *Cross-sectional study—*Report numbers of outcome events or summary measures | *NA* |  |
| Main results | 16 | (*a*) Give unadjusted estimates and, if applicable, confounder-adjusted estimates and their precision (eg, 95% confidence interval). Make clear which confounders were adjusted for and why they were included | 10-15 | Basic analysis is given as is required for this kind of study. |
|  |  | (*b*) Report category boundaries when continuous variables were categorized | 14-15 | Table 2 and Table 3 |
|  |  | (*c*) If relevant, consider translating estimates of relative risk into absolute risk for a meaningful time period | NA |  |

Continued on next page

| Other analyses | 17 | Report other analyses done—eg analyses of subgroups and interactions, and sensitivity analyses | 16 | There was very weak evidence of a difference in seropositivity by sex, with seropositivity slightly higher in males (9.9%) as compared to females (7.2%), (p=0.08). There was a strong association between increasing age and seroprevalence (z=6.29; p<0.001). |
| --- | --- | --- | --- | --- |
| Discussion | | | | |
| Key results | 18 | Summarise key results with reference to study objectives | 16-20 | The parasitological and entomological findings suggest there is zone of perennial high transmission, around the sentinel village of Makouopsap.  To conclude, both parasitological and entomological findings confirm an ongoing transmission of O. volvulus in the Massangam HD despite over 18 years of uninterrupted annual mass distribution of ivermectin. The study was able to refine the boundaries of a high zone of transmission (approximately 12km) around the sentinel site of Makouopsap and facilitated by productive breeding sites on the River Nja and River Mbam. |
| Limitations | 19 | Discuss limitations of the study, taking into account sources of potential bias or imprecision. Discuss both direction and magnitude of any potential bias | 17, 20 | The lack of standardisation of traps and human collections used makes it more difficult to compare data across time periods.  There were issues of high rates of refusal for skin snip biopsies in children in the study communities, which resulted in a change in inclusion criteria after the start of the data collection. The low response rate in children for skin snip biopsies were offset by the higher response rate in the use of the Ov-16 rapid test, which was more useful in determining historical transmission dynamics in the area. The skin snips were also not weighed, which would have provided a better indication of CMFL, although efforts were made to standardize the skin biopsy taken so the variability in weight should not have been large. |
| Interpretation | 20 | Give a cautious overall interpretation of results considering objectives, limitations, multiplicity of analyses, results from similar studies, and other relevant evidence | 16-20 | As above |
| Generalisability | 21 | Discuss the generalisability (external validity) of the study results | NA |  |
| Other information | |  | | |
| Funding | 22 | Give the source of funding and the role of the funders for the present study and, if applicable, for the original study on which the present article is based | 21 | The funds for the execution of this study were received from Sightsavers. All number of co-authors work for Sightsavers and were involved at all stages of the study design, data collection and analysis, decision to publish preparation of the manuscript. |

*Give information separately for cases and controls in case-control studies and, if applicable, for exposed and unexposed groups in cohort and cross-sectional studies.

**Note:** An Explanation and Elaboration article discusses each checklist item and gives methodological background and published examples of transparent reporting. The STROBE checklist is best used in conjunction with this article (freely available on the Web sites of PLoS Medicine at http://www.plosmedicine.org/, Annals of Internal Medicine at http://www.annals.org/, and Epidemiology at http://www.epidem.com/). Information on the STROBE Initiative is available at www.strobe-statement.org.
